# Resistance‐exercise‐induced stress intervenes in TGF‐β signaling by cooperatively downregulating nuclear αB‐crystallin and SMAD4 in human skeletal muscle fibers

**DOI:** 10.1111/febs.70468

**Published:** 2026-02-23

**Authors:** Kirill Schaaf, Cassandra Duda, Tihomir Kostov, Patrick Diel, Käthe Bersiner, Wilhelm Bloch, Sebastian Gehlert, Daniel Jacko

**Affiliations:** ^1^ Department of Molecular and Cellular Sports Medicine, Institute of Cardiovascular Research and Sports Medicine German Sport University Cologne Cologne Germany; ^2^ Department for the Biosciences of Sports, Institute of Sports Science University of Hildesheim Hildesheim Germany

**Keywords:** CRYAB, HSPB5, muscle, nuclei, resistance exercise, SMAD4, TGF‐β

## Abstract

Alpha‐crystallin B chain (αB‐crystallin; CRYAB) helps to maintain proteostasis following cellular stress. Additionally, recent studies in fibroblasts have shown its function in the transforming growth factor beta (TGF‐β) pathway, where it stabilizes mothers against decapentaplegic homolog 4 (SMAD4) in the nucleus, enabling target gene transcription. Specifically in skeletal muscle fibers (SkMFs), TGF‐β/SMAD4 signaling via myostatin regulates fiber size by inducing growth inhibitors and suppressing the serine/threonine‐protein kinase (mTOR) pathway. Conversely, resistance exercise (RE) promotes muscle growth and should therefore affect CRYAB/SMAD4 interaction. However, in human SkMFs, CRYAB's nuclear localization and role in TGF‐β/SMAD4 signaling, at rest or after RE, remain undescribed. Therefore, we first validated CRYAB's nuclear localization by small interfering RNA (siRNA)‐mediated *CRYAB* silencing in C2C12 cells (mouse skeletal muscle myoblast cell line) and in human SkMFs via subcellular fractionation and confocal microscopy. To determine the effect of RE on this pathway, skeletal muscle biopsies were taken at rest and 60 min post‐RE and analyzed for nuclear localization and phosphorylation of CRYAB and SMAD4, CRYAB/SMAD4 colocalization, and anabolic signaling. CRYAB was localized to C2C12 myoblast nuclei during proliferation but disappeared early during differentiation and was absent in nuclei of myotubes. In contrast, CRYAB was present in nuclei of resting human SkMFs. However, acute RE reduced total CRYAB in SkMF nuclei and increased its phosphorylation at serine 59, which likely promotes its export. Acute RE also reduced both nuclear and cytoplasmic SMAD4 while enhancing anabolic signaling by mTOR and small ribosomal subunit protein eS6 (rpS6) phosphorylation. We conclude that CRYAB and SMAD4 interact in nuclei of human SkMFs and have a coordinated regulation in response to RE‐induced mechanical stress, revealing a new perspective on a yet‐undescribed mechanism contributing to skeletal muscle adaptation.

## Graphical abstract

Alpha‐crystallin B chain (CRYAB) has been reported to stabilize mothers against decapentaplegic homolog 4 (SMAD4) in transforming growth factor‐β (TGF‐β) signaling, enabling target gene transcription. We show nuclear CRYAB–SMAD4 interaction for the first time in human skeletal muscle fibers and its regulation by exercise. At rest, nuclear CRYAB/SMAD4 indicates stabilized transcriptional complexes and expression of genes preserving fiber size. Resistance exercise disrupts stabilization, limits fiber size negative regulators, and permits growth.

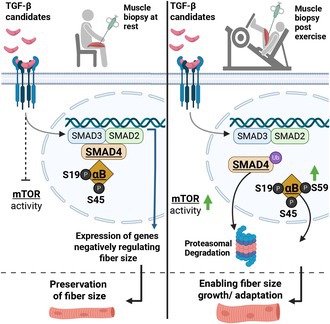

**Abbreviations**
AKT: RAC serine/threonine‐protein kinase
BSA: Bovine serum albumin
CEB: Cytoplasmic extraction buffer
Cht: Chromatin‐bound
CRYAB: Alpha‐crystallin B chain
CS: Cytosol
F‐IHC: Fluorescence immunohistochemistry
FOXO1/3: Forkhead box protein O1/3
GAPDH: Glyceraldehyde‐3‐phosphate dehydrogenase
h: Hours
H3: Histone H3
HDAC1: Histone deacetylase 1
LSM: Laser scanning microscope
MAP: kinase‐activated protein kinase 2
Mb: Membrane
MEB; MHC: Myosin heavy chain, MK2
mTOR: Serine/threonine‐protein kinase mTOR
MuRF1: E3 ubiquitin‐protein ligase TRIM63
Myf5: Myogenic factor 5
Np: Nucleoplasm
p38 MAPK: Mitogen‐activated protein kinase 11–14
Pel: Pellet
qPCR: quantitative PCR
RE: Resistance exercise
ROI: Region of interest
rpS6: Small ribosomal subunit protein eS6
SC‐35: serine/arginine‐rich splicing factor SC35
SDHA: Succinate dehydrogenase [ubiquinone] flavoprotein subunit
Ser: Serine
siRNA: Small interfering RNA
SkMFs: Skeletal muscle fibers
SMAD: Mothers against decapentaplegic homolog
SMN: Survival motor neuron protein
TGF‐β: Transforming growth factor‐beta proprotein
TIF1γ: E3 ubiquitin‐protein ligase TRIM33
WGA: Wheat germ agglutinin

## Introduction

Skeletal muscle is a highly adaptive tissue that accounts for about 40% of the human body mass. Preservation of skeletal muscle mass is pivotal for whole body health, while its loss is associated with various diseases like cancer, diabetes, or sarcopenia [1]. Resistance exercise (RE) is a well‐established strategy to promote muscle hypertrophy and health promoting adaptations for the entire organism. It activates signaling networks responsible for protein synthesis and myofiber growth, particularly the AKT/mTOR‐pathway (RAC‐alpha serine/threonine‐protein kinase/serine/threonine‐protein kinase mTOR) [2, 3, 4] and downregulates pathways that promote protein degradation or hinder synthesis, like TGF‐β‐family members (transforming growth factor beta) [1, 5]. Especially the myokine myostatin [6, 7], but also TGF‐β1 (transforming growth factor beta‐1 proprotein) [8, 9], act as negative regulators of muscle mass. Their receptor binding facilitates activation of receptor‐regulated SMAD (mothers against decapentaplegic homolog) proteins (SMAD2/3), which then bind with the key mediator SMAD4 [10, 11], enabling the complex to enter the nucleus [11, 12]. Transcription and activity of ubiquitin proteolytic system candidates, like MuRF1 (E3 ubiquitin‐protein ligase TRIM63), FOXO1/3 (Forkhead box protein O1/3), or Atrogin‐1 (F‐box only protein 32), become upregulated, while the expression of growth‐promoting genes, such as *MyoD1* [13, 14, 15, 16], and activation of the anabolic AKT/mTOR‐pathway are reduced [1, 17, 18].

In the nucleus, a key regulator of SMAD4 is the E3 ubiquitin‐protein ligase TRIM33 (TIF1γ). TIF1γ monoubiquitinates SMAD4, leading to its dissociation from SMAD2/3 and nuclear export back into the cytosol where it is subjected to proteasomal degradation [19, 20, 21]. Recently, CRYAB (Alpha‐crystallin B chain), a small heat shock protein, was identified as a novel regulator of SMAD4 sequestration in the nucleus. It was demonstrated that, in fibrocytes, CRYAB stabilizes nuclear SMAD4 by compromising its interaction with TIF1γ, thus preventing its ubiquitination and nuclear export [22, 23, 24, 25]. This finding is intriguing for two reasons: (a) It establishes a previously unknown link between CRYAB and TGF‐β signaling, and (b) it highlights an underexplored nuclear function of CRYAB.

CRYAB is traditionally known for its chaperone activity [26, 27], particularly in skeletal muscle, where it protects cytoskeletal proteins like desmin from stress‐induced damage [28, 29, 30, 31]. CRYAB has three main phosphorylation sites: Serine (Ser) 19, Ser45, and Ser59 [32], which are important for its function [33, 34, 35, 36]. Our group and others have shown that CRYAB becomes rapidly phosphorylated at Ser59 only upon resistance exercise‐induced stress but not following endurance exercise [37, 38, 39, 40]. However, data on CRYAB's nuclear localization, specifically in human skeletal muscle and as a response to growth‐promoting RE, are not available. Previous reports in C2C12 myoblasts [41] and HeLa cells [42, 43, 44] suggest that it localizes in nuclear speckles and that nuclear import of CRYAB depends on its phosphorylation at Ser59 [45]. Nevertheless, its function in the nucleus remains poorly understood. Furthermore, previous studies in muscle cells relied primarily on immunohistochemical analysis without additional biochemical validation [41, 46]. To date, no data exist concerning a nuclear CRYAB localization in human SkMFs or its potential interaction with SMAD4 in nuclei.

Thus, the objective of this study was to: (a) Validate the specificity of nuclear CRYAB localization using siRNA‐mediated gene silencing in C2C12 cells, (b) Investigate its presence in human SkMFs through subcellular fractionation and fluorescence immunohistochemistry (F‐IHC), (c) Examine whether nuclear CRYAB and SMAD4 colocalize, and (d) Assess how they respond to contraction‐induced stress by acute RE.

Our findings demonstrate that CRYAB is localized in nuclei of human SkMFs and phosphorylated at Ser19 and Ser45, but hardly at Ser59.

RE‐induced stress induces a reduction of the nuclear CRYAB content and a concomitant increase in its phosphorylation at Ser59 (_p_CRYAB^Ser59^), which might trigger the reduction of CRYAB via nuclear export. This is accompanied by a concurrent decrease in SMAD4 levels in both the nuclei and cytoplasm as well as the phosphorylation of mTOR and rpS6 (small ribosomal subunit protein eS6) as key regulators of protein synthesis. These results strongly indicate that both CRYAB and SMAD4 interact in nuclei of human SkMFs, where CRYAB likely stabilizes SMAD4 and facilitates TGF‐β signaling. RE disrupts this interaction, temporarily attenuating TGF‐β signaling to promote SkMF adaptation.

## Results

### CRYAB is localized in nuclei of C2C12 myoblasts and of human SkMFs but is absent in nuclei of differentiated myotubes

**Fig. 1:**
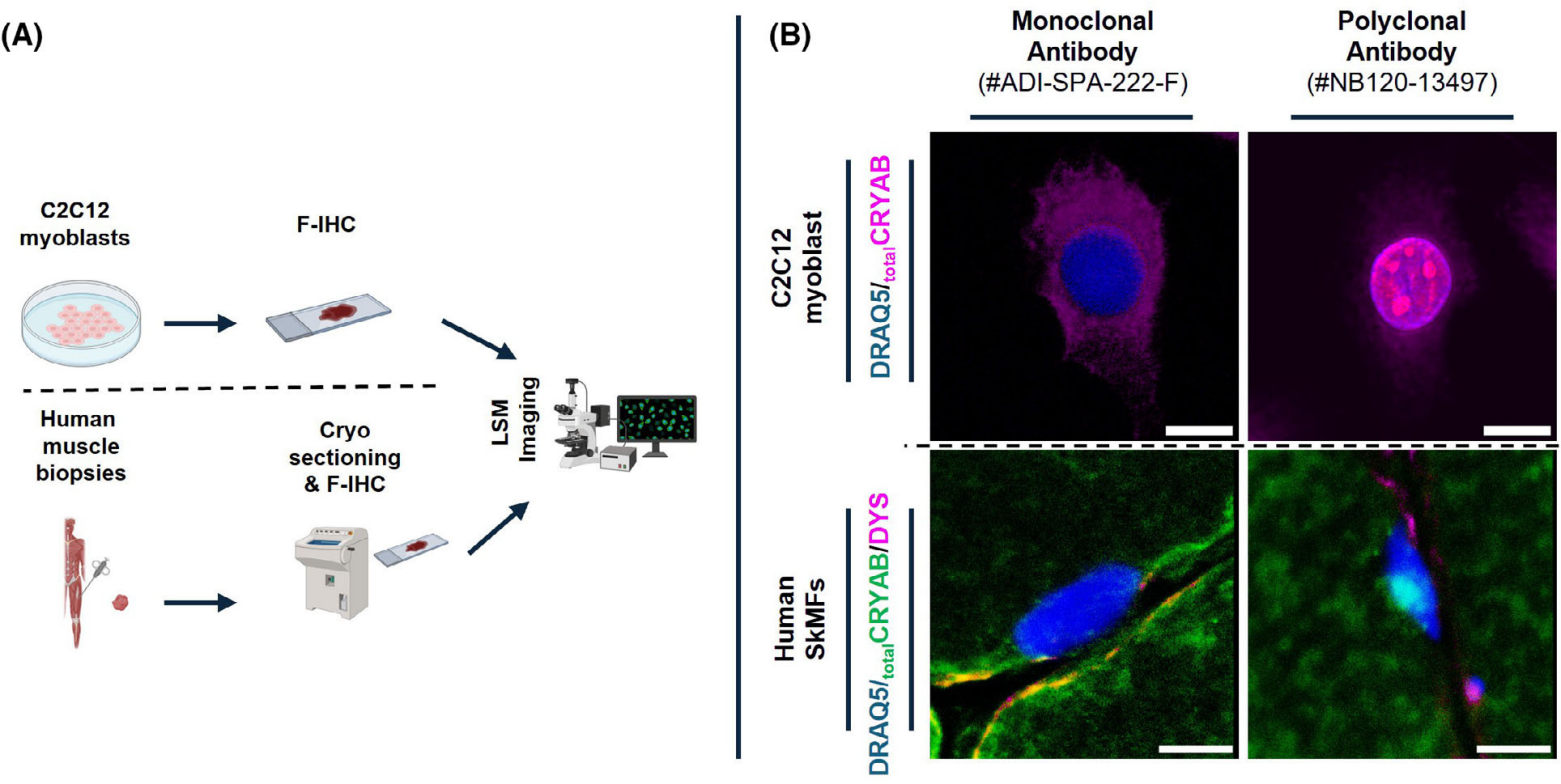
Antibody‐dependent differences in the detection of a nuclear signal from CRYAB. (A) Experimental procedure. C2C12 myoblasts and human muscle biopsies were stained by fluorescence immunohistochemistry (F‐IHC) for CRYAB and imaged by a laser scanning microscope (LSM). (B) F‐IHC in C2C12 myoblasts (upper panel; *n* = 3) and human skeletal muscle fibers (SkMFs, lower panel, *n* = 10). Staining of C2C12 myoblasts: total CRYAB; monoclonal and polyclonal (magenta), DRAQ5 (blue). Staining of human SkMFs: total CRYAB; monoclonal and polyclonal (green), Dystrophin (magenta), DRAQ5 (blue). Scale bars: 10 μm for C2C12 myoblast images, 5 μm for human SkMFs images. Image B, bottom right side, has been reused in Fig. 4B.

CRYAB is a well‐characterized chaperone in skeletal muscle cells and fibers [28]. Only a few *in vitro* studies have examined its presence and function in the nuclear compartment, and those that did were based mainly on F‐IHC data [41, 46]. Furthermore, not every available antibody detects CRYAB in nuclei [43] (Fig. 1A,B), what in sum may leave the specificity of the observed signal open to question [44, 47].

Thus, as a first step, we conducted a proof‐of‐concept experiment to extensively validate the specificity of the nuclear CRYAB signal in C2C12 cells and human SkMFs.

**Fig. 2:**
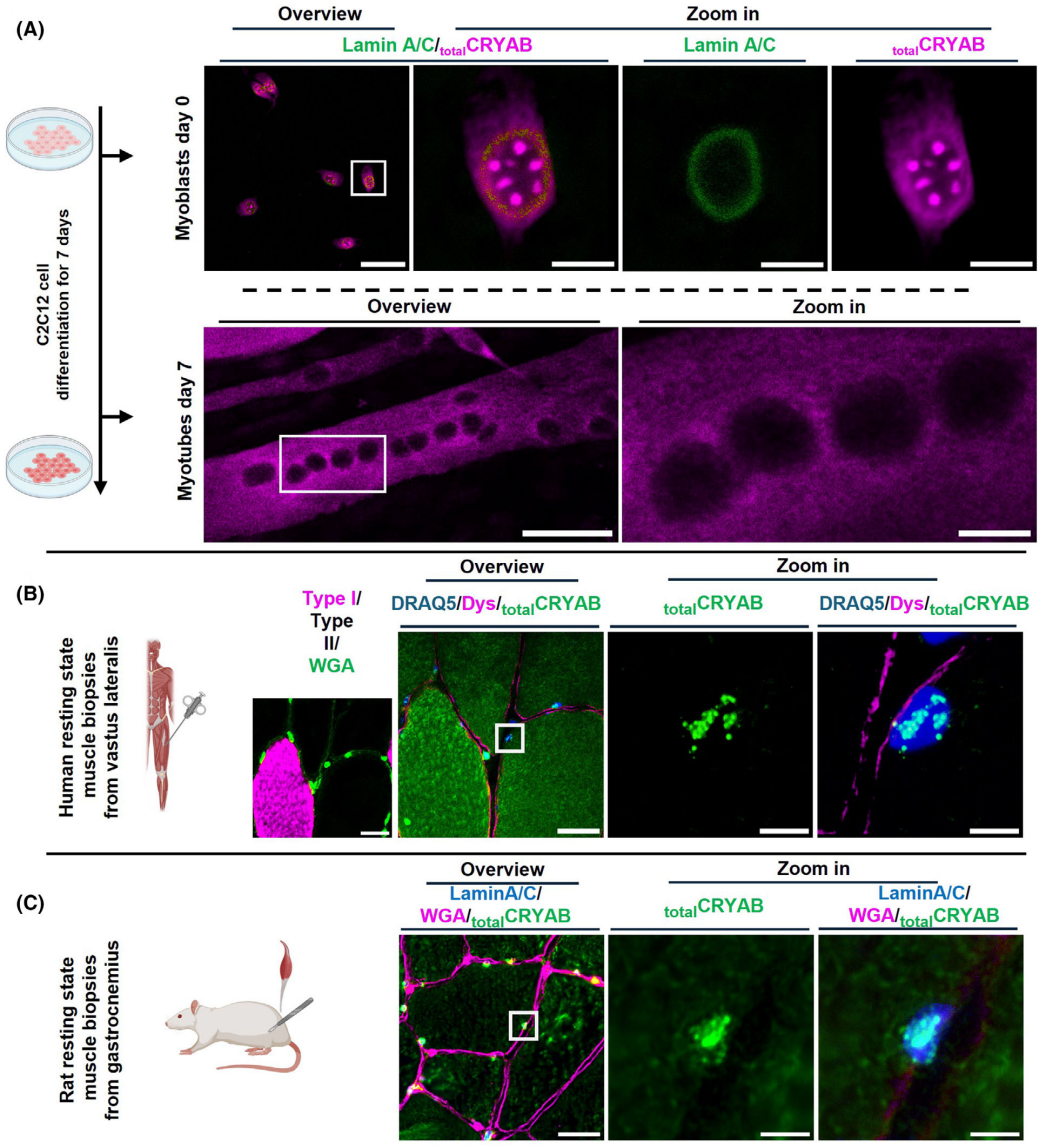
Determination of nuclear CRYAB signal in C2C12 myoblasts and human skeletal muscle fibers. (A) Fluorescence immunohistochemistry (F‐IHC) in C2C12 myoblasts (upper panel; *n* = 3) and myotubes at day seven of differentiation (lower panel; *n* = 3). Staining: total CRYAB: (magenta), Lamin A/C (green). Scale bars: 50 μm for overview images, 10 μm for zoomed‐in images. Nuclei in myotubes are unstained. (B) F‐IHC images of human skeletal muscle cross‐sections. Vastus lateralis muscle biopsies were taken from resting muscle (*n* = 10). Staining from left to right image: 1. Fiber types (Type I: magenta, type II: unstained), membranes (Wheat Germ Agglutinin [WGA]: green); 2, 3, and 4. CRYAB (green), membranes (Dystrophin; magenta), nuclei (DRAQ5, blue). (C) F‐IHC images of rat gastrocnemius muscle cross‐sections (*n* = 3). Staining: membranes (Wheat Germ Agglutinin [WGA]: magenta); CRYAB (green); nuclei (Lamin A/C, blue). Scale bars: 30 μm for overview images, 5 μm for zoomed‐in images.

F‐IHC staining was positive in nuclei of C2C12 myoblasts (Fig. 2A, upper panel); however, no signal was apparent in myotubes differentiated for seven days (Fig. 2A, lower panel), which is consistent with previous studies [41, 46]. On the other hand, nuclei of human skeletal muscle fibers, taken at rest, show a clear signal with the CRYAB antibody (Fig. 2B), which is demonstrated for the first time.

Additionally, to investigate whether nuclear CRYAB is specific to human SkMFs or represents a conserved feature of adult mammalian muscle, we stained rat gastrocnemius muscle sections for total CRYAB (Fig. 2C). Similar to human muscle, these sections showed a clear nuclear signal, indicating that CRYAB localizes to myonuclei across species.

**Fig. 3:**
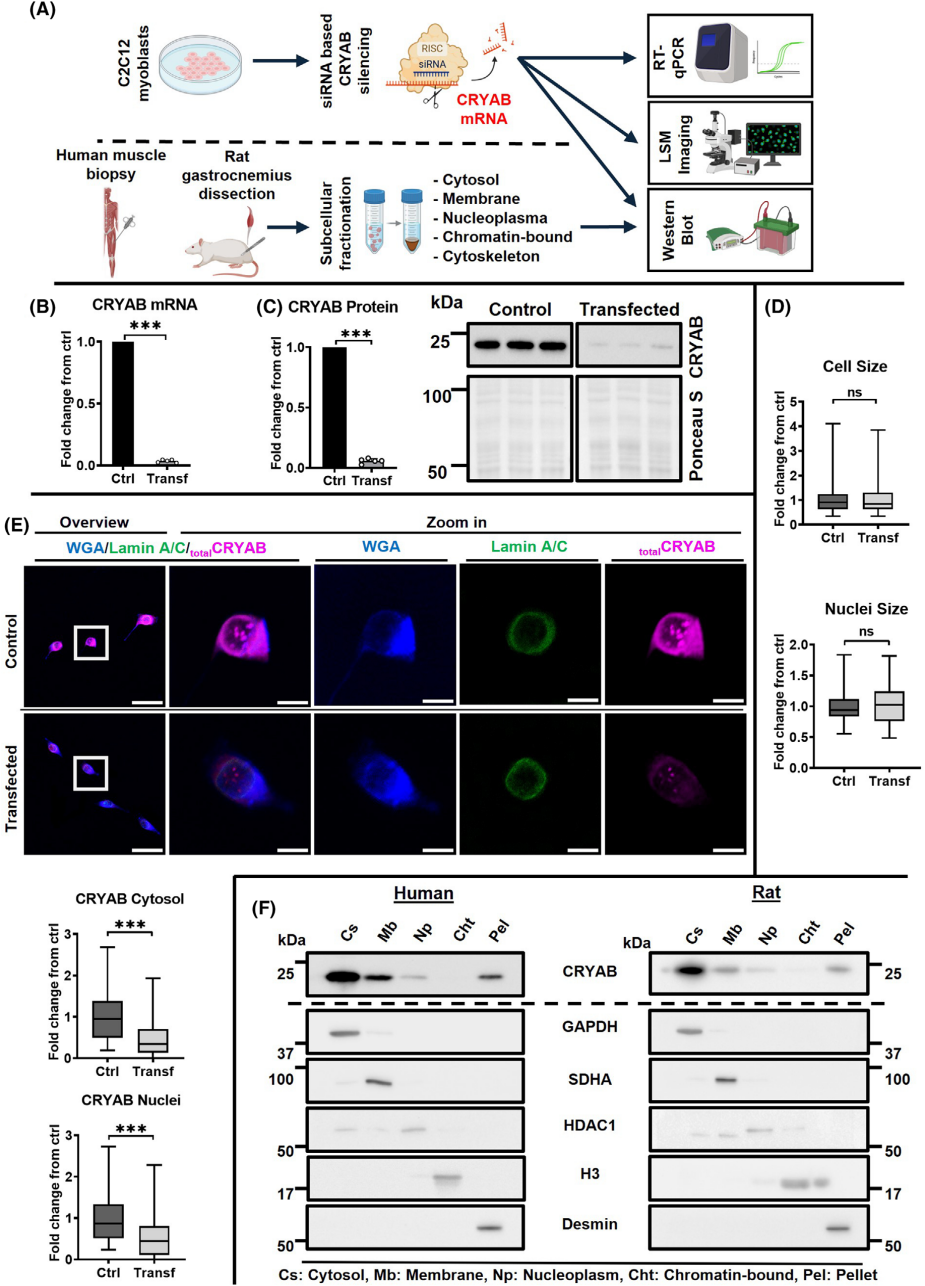
Validation of nuclear CRYAB signal in C2C12 myoblasts and human skeletal muscle fibers (A) Experimental procedure. CRYAB was silenced in C2C12 myoblasts via siRNA. RT‐qPCR, fluorescence immunohistochemistry (F‐IHC), and western blotting (WB) were performed on transfected and control samples. Additionally, resting human skeletal muscle biopsies were collected from vastus lateralis. One part was cryo‐sectioned and used for F‐IHC, then imaged with a laser scanning microscope (LSM). Another part was subjected to subcellular fractionation into cytosolic, membrane, nucleoplasmic, chromatin‐bound, and pellet or cytoskeletal proteins, followed by western blot analysis. (B) RT‐qPCR (*n* = 5) and (C) western blot analysis of CRYAB mRNA expression levels from CRYAB siRNA‐transfected (Transf) and control (Ctrl) C2C12 myoblasts (*n* = 5). mRNA and protein levels of control samples were normalized to one. Error bars show +SEM. (C) Right panel: Representative CRYAB band images and corresponding Ponceau S staining as loading control (each three technical replicates). (D) Quantification of cell and nuclear size of control and transfected C2C12 myoblasts (*n* = 3). Data represent fold changes relative to control cells. For this, all numbers of control and transfected cells were divided by the mean of control. At least 140 (cell size) or 90 (nuclear size) individual cells per condition were analyzed. Data are presented as box plots showing median (center line), interquartile range (box), and whiskers indicating the minimum and maximum values. ns = not significant. (E) Quantification of CRYAB staining intensity in the cytosol and nuclei of the control and CRYAB siRNA‐transfected C2C12 myoblasts (*n* = 3). Upper panel: Representative F‐IHC images of CRYAB (magenta), nuclei (Lamin A/C; green), and membranes/cell body (Wheat Germ Agglutinin [WGA], blue) from control and transfected C2C12 myoblasts. Scale bars: 30 μm for overview images, 5 μm for zoomed‐in images. Lower panel: Box plots of CRYAB staining intensity within cytosolic and nuclear compartments. Data represent fold changes relative to control cells. For this, all numbers of control and transfected cells were divided by the mean of control. At least 90 individual cells per condition were analyzed. Data are presented as box plots showing median (center line), interquartile range (box), and whiskers indicating the minimum and maximum values. (F) Subcellular fractions of human skeletal muscle biopsies described in (A) (left, *n* = 2) and gastrocnemius muscle of the rat (right, *n* = 3). Representative western blot images of CRYAB and fraction purity controls always show the results of the same person or animal. Corresponding Ponceau S stainings can be found in the supplementals (Fig. S1C). Statistical analysis was performed using two‐tailed unpaired *t*‐tests. ***P < 0.001; ns = not significant.

Next, to validate the staining‐ and antibody specificity, a siRNA‐based *CRYAB* knockdown in C2C12 myoblasts was performed, with subsequent RT‐qPCR, WB, and F‐IHC analyses (Fig. 3A). To verify successful knockdown, RT‐qPCR and WB were carried out. RT‐qPCR revealed the downregulation of *CRYAB* mRNA expression in transfected cells as compared to control (Figs 3B, S1), which was supported at the protein level by a near disappearance of the 22 kDa CRYAB band (Fig. 3C). Thus, both analyses supported a highly effective silencing of CRYAB.

As silencing of small heat shock proteins was shown to influence cell shape and morphology [48], which might impact our following comparisons, we measured cell and nuclear size and determined that *CRYAB* silencing had no effect on both parameters (Fig. 3D).

After confirming that *CRYAB* silencing was successful, we determined whether the nuclear signal detected via F‐IHC is specific for CRYAB (Fig. 3E). The staining intensity of cytosolic and especially nuclear CRYAB signal was significantly reduced in transfected C2C12 myoblasts compared to control, demonstrating the specificity of the nuclear CRYAB signal.

Finally, to verify the presence and specificity of nuclear CRYAB in human and rat SkMFs, muscle tissue fractionation was performed, generating five fractions: (a) Cytosolic proteins, (b) Membrane proteins, (c) Nucleoplasmic proteins, (d) Chromatin‐bound proteins, and (e) Pellet/cytoskeletal proteins (Fig. 3F). Further, the purity of each fraction was validated by respective proteins. According to previous reports, in resting or unstressed human muscle, the sarcoplasm contains the biggest amount of CRYAB. The protein is also found in the membrane fraction, with only a small portion associated with the cytoskeleton [37, 38, 49, 50]. At the same time, we show for the first time for human and rat SkMFs that a portion of the protein is also located in the nucleoplasm but not chromatin‐associated, and thus supporting the observations by den Engelsman *et al*. [44]. They have shown that DNase I and RNase A treatment of cells did not disrupt CRYAB's speckle‐like assembly, indicating that intact DNA or RNA is not necessary for the spatial organization of the protein and chromatin binding is highly unlikely.

### Nuclear CRYAB localization in human SkMFs is sensitive to RE‐induced stress

**Fig. 4:**
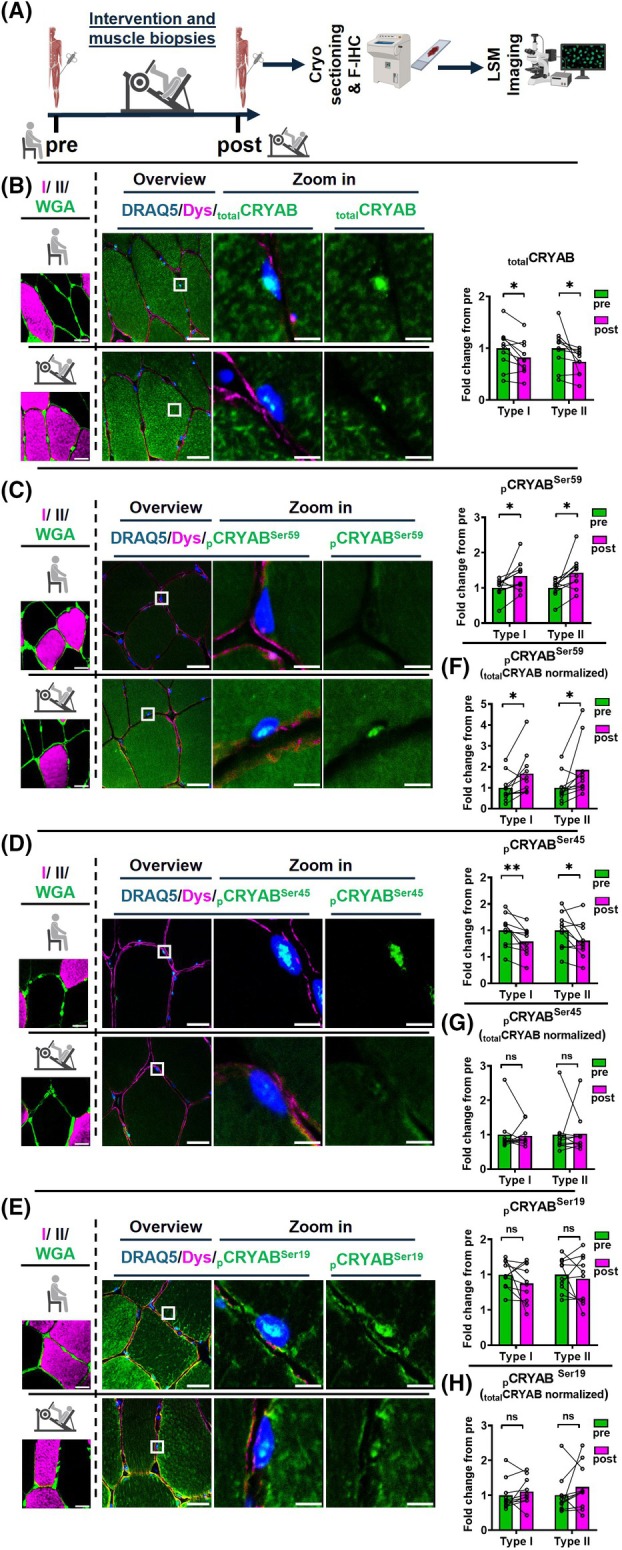
The Effect of resistance exercise (RE) on CRYAB content and phosphorylation in nuclei of human skeletal muscle fibers. (A) Experimental procedure. Human skeletal muscle biopsies were collected before (pre) and one hour after (post) a single bout of RE. Biopsies were cryo‐sectioned and used for fluorescence immunohistochemistry (F‐IHC), then imaged with a laser scanning microscope (LSM). (B–E) Quantification of nuclear total (B) and phospho (C–E) CRYAB signal pre‐ and post‐RE (*n* = 10). Left panel: Representative F‐IHC images. Small images left: Fiber type staining (Type I: magenta; type II: unstained; membranes: green [WGA]). Remaining images: CRYAB (green), membranes (Dystrophin; magenta), nuclei (DRAQ5, blue). Scale bars: 30 μm for overview images, 5 μm for zoomed‐in images. Representative images of pre and post‐RE always show the results of the same person. Right panel: Quantification of staining intensity of indicated targets in nuclei of type I and type II muscle fibers. For quantification, an average of 126.4 ± 25.7 nuclei per subject and time point (mean ± SD) were analyzed, and per subject means were used for further analyses. (F–H) Quantifications of nuclear phospho CRYAB with signals normalized to nuclear total CRYAB (*n* = 10). Data (B–H) represent fold changes relative to pre. For this, individual subject means of pre and post were divided by the overall mean of pre. Bars represent mean values, individual data points are shown, and paired data from the same subject are connected by lines. Statistical analysis was performed using two‐tailed paired *t*‐tests. *P < 0.05; **P < 0.01; ***P < 0.001; ns = not significant. Image B, middle, has been reused in Fig. 1B, bottom right side.

CRYAB is highly inducible by stress [51]. In human skeletal muscle, sarcoplasmic CRYAB becomes rapidly phosphorylated upon RE and then associates with unfolding cytoskeletal proteins to prevent their damage [37, 38, 39, 40, 50]. However, to date, it was not known whether nuclear CRYAB in human SkMF nuclei is also subjected to stress‐induced changes in its localization, abundance, or phosphorylation. Accordingly, first, we applied lower limb RE to subjects and took muscle biopsies from the vastus lateralis at rest (pre) and one hour post‐RE (post), followed by F‐IHC and LSM imaging (Fig. 4A). As skeletal muscle is comprised of various fiber types, each with distinct contractile properties, responding differently to stress and adapting in a fiber type‐specific manner [52, 53, 54, 55, 56], we also performed a fiber‐type‐specific analysis, differentiating between type I and IIA fibers.

We show that nuclear content of total CRYAB is attenuated following RE in both type I and type IIA fibers (Fig. 4B). Moreover, RE significantly impacts the phosphorylation of CRYAB: phosphorylation at Ser59 (Fig. 4C) increases, while it decreases at Ser45 (Fig. 4D), with no change observed for Ser19 (Fig. 4E). However, when accounting for total CRYAB attenuation after RE and normalizing the phosphorylation signal to total CRYAB content, only an upregulation of Ser59 is revealed significant (Fig. 4F–H). In general, neither pre‐ nor post‐RE (Fig. S2A) was there a difference in CRYAB phosphorylation levels between fiber types, except for _p_CRYAB^Ser45^ at resting state (Fig. S2B).

### Ser59 phosphorylation accompanies nuclear depletion of CRYAB in human SkMFs and C2C12 cells, suggesting a role in nuclear export

**Fig. 5:**
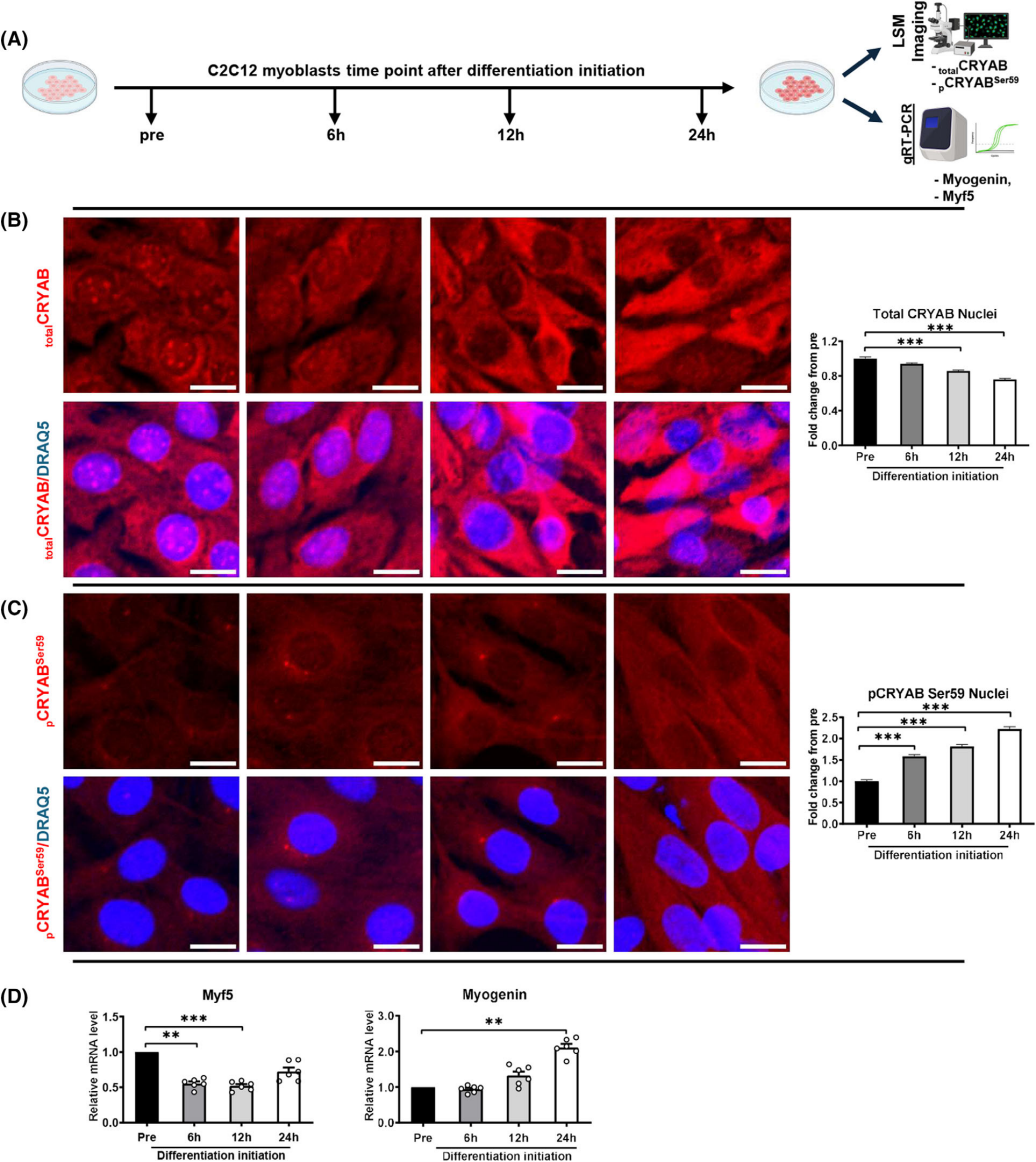
Behavior of _total_CRYAB and _p_CRYAB^Ser59^ in C2C12 myoblasts within 24 hours upon differentiation initiation. (A) C2C12 myoblasts were differentiated into myotubes for 24 h. Cells were fixed prior to substitution from proliferation to differentiation medium (pre) as well as after 6, 12, and 24 h, followed by an immunofluorescence staining for (B) _total_CRYAB and (C) _p_CRYAB^Ser59^ (red) and nuclei (DRAQ5, blue). Scale bars represent 50 μm for overview images and 15 μm for zoomed‐in images (*n* = 3). Representative images of different time points always show the results of the same experiment. For quantification, approximately 250 nuclei per time point were analyzed. In parallel, total RNA was extracted at each time point and (D) analyzed by RT‐qPCR for *Myogenic factor 5* (*Myf5*) and *Myogenin* (*n* = 6). Data are presented as means + SEM (B, C, and D). Statistical analysis was performed using the Kruskal–Wallis test and Dunn's test for multiple comparisons with “Pre” as the control column. **P < 0.01, ***P < 0.001.

As serine 59 was the only significantly upregulated site at CRYAB when normalized for total protein amount, and as CRYAB function and localization relies on phosphorylation [33, 34, 35, 36], we assumed that this phosphorylation site might be involved in its nuclear export. To verify this, we used the aforementioned observation that C2C12 cells lose their nuclear CRYAB signal due to differentiation into myotubes (Fig. 2A). We analyzed the nuclear CRYAB and _p_CRYAB^S59^ signals in a time series from the proliferative stage (pre) up to 24 h upon differentiation initiation. Additionally, we tested for *Myogenin* and *Myf5 (Myogenic factor 5)* expression to specify the differentiation phase of nuclear CRYAB disappearance (Fig. 5A).

After only six hours following the substitution from proliferation to differentiation medium, the appearance of the nuclear CRYAB signal began to change from a speckle‐like pattern to a more diffuse distribution (qualitative image assessment, Fig. 5B, left panel). The quantification shows a decline in the nuclear CRYAB signal after 12 and 24 h (Fig. 5B, right panel) whereby it is no longer recognizable after 7 days of differentiation at the latest (Fig. 2B, lower panel).

These reductions in total CRYAB were accompanied by an increase in _p_CRYAB^S59^ in the nuclei (Fig. 5C). We detected this coincidence already in human SkMFs following RE (Fig. 4B,C), which underpins the relevance of this phosphorylation site for nuclear export of CRYAB.

Furthermore, the expression of *Myf5*, which was downregulated at six and 12 h, and *Myogenin*, which was upregulated at 24 h (Fig. 5D), revealed that nuclear CRYAB downregulation starts approximately at the onset of the early differentiation phase and is almost completed with the onset of the myoblast fusion phase at 24 h.

Thus, during this time frame, nuclear CRYAB may no longer be required—or conversely, its disappearance from the nucleus may be essential for the proceeding of the phases.

### SMAD4 colocalizes with CRYAB in nuclei of human SkMFs at rest. RE Decreases the nuclear and cytosolic SMAD4 abundance with simultaneous upregulation of pro‐ anabolic signaling

Bellaye *et al*. [23] have shown that CRYAB is involved in the TGF‐β1‐pathway and is responsible for SMAD4 sequestration in nuclei of fibroblasts [24, 25]. However, in the nuclei of human SkMFs, SMAD4/CRYAB colocalization has not yet been determined, neither in the resting state nor following RE.

**Fig. 6:**
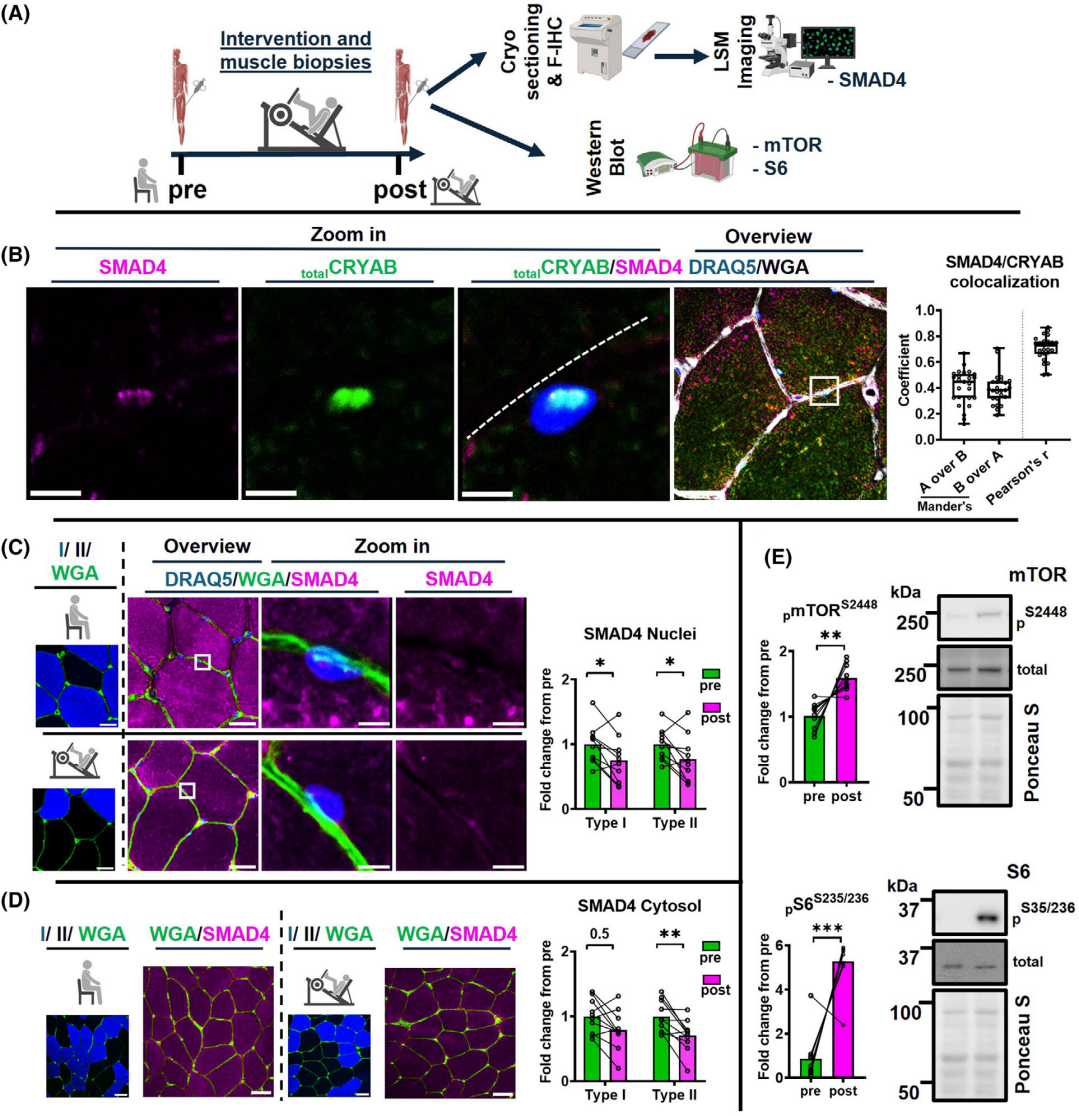
CRYAB and SMAD4 colocalization in nuclei of human skeletal muscle fibers and the effect of resistance exercise (RE) on nuclear and sarcoplasmic SMAD4 content. (A) Experimental procedure. Human skeletal muscle biopsies were collected before (pre) and one hour after (post) a single bout of RE. One part was cryo‐sectioned and used for fluorescence immunohistochemistry (F‐IHC), then imaged with a laser scanning microscope (LSM) to assess SMAD4. Another part was subjected to western blot (WB) analysis to assess protein synthesis of related anabolic targets (mTOR, rpS6). (B) Analysis of total CRYAB/SMAD4‐colocalization in nuclei of human SkMFs at rest. Left panel: Representative F‐IHC images. Staining: CRYAB (green), SMAD4 (magenta), nuclei (DRAQ5, blue) membranes (Wheat Germ Agglutinin [WG], white; indicated as dashed line in zoomed‐in image). Scale bars: 30 μm for overview images, 5 μm for zoomed‐in images. Right panel: Quantification shows Manders' and Pearson's correlation coefficients for SMAD4 and CRYAB colocalization in 25 individual nuclei. Data are presented as box plots showing median (center line), interquartile range (box), individual data points, and whiskers indicating the minimum and maximum values. (C) Quantification of nuclear (*n* = 10) and cytosolic (*n* = 10) (D) SMAD4‐signal pre‐ and post‐RE. Left panel: Representative F‐IHC images. Small images left: Fiber type staining (Type I: blue; type II: unstained; membranes: green [WG]). Remaining images: SMAD4 (magenta), membranes (WG; green), nuclei (DRAQ5, blue). Scale bars: 30 μm for overview images, 5 μm for zoomed‐in images. Representative images of pre‐ and post‐RE always show the results of the same person. Right panel: Quantification of staining intensity of SMAD4 in nuclei of type I and type II muscle fibers. For quantification, an average of 80.8 ± 23.0 nuclei and 93.6 ± 34.23 fibers per subject and time point (mean ± SD) were analyzed, and per subject means were used for further analyses. (E) WB analysis of protein synthesis‐related targets pre‐ and post‐RE (*n* = 10). Upper panel: total and phosphorylated mTOR at serine 2448. Lower panel: total and phosphorylated ribosomal protein S6 at serine 235/236. For each protein corresponding representative phospho and total WB‐bands and Ponceau S images are always from the same person. mTOR and S6 were run on the same gel. Data (C, E) are presented as fold changes relative to pre. For this, individual subject means of pre and post were divided by the overall mean of pre. Bars represent mean values, individual data points are shown, and paired data from the same subject are connected by lines. Statistical analysis was performed using two‐tailed unpaired *t*‐tests. *P < 0.05; **P < 0.01; ***P < 0.001.

Thus, we next tested the colocalization of both proteins in muscle samples taken from subjects at rest and performed F‐IHC analysis (Fig. 6A). We obtained a moderate to high Pearson's coefficient (*r* = 0.71) but a moderate to low Manders' coefficient (Fig. 6B; M1 = 0.41 SMAD4 overlapping CRYAB; M2 = 0.39 CRYAB overlapping SMAD4). This suggests that both proteins do not perfectly overlap and colocalize, which could occur when there is a dynamic interaction between the two proteins, meaning colocalization is only partial at any given time point. This is plausible, as SMAD4 does not reside constantly in the nucleus but continuously undergoes a cyto‐nucleoplasmic shift, which even occurs independently from TGF‐β stimulation [57, 58]. Nevertheless, the data indicate that at least a portion of both proteins is spatially connected within the nuclei of human SkMFs without RE stimulation.

In fibroblasts, nuclear SMAD4 sequestration by CRYAB promotes TGF‐β1 signaling, which has a pro‐fibrotic effect in this cell type [23, 24, 25]. However, TGF‐β candidates act very cell‐specifically and their function differs between fibroblasts and SkMFs. In SkMFs, TGF‐β1 and myostatin exhibit mainly an inhibitory effect on fiber size and repair [6, 7, 8, 9]. RE, on the other hand, promotes fiber size growth [59] and induction of repair mechanisms [60]. Consequently, RE should affect CRYAB and SMAD4 regulation. Specifically, since RE reduces nuclear CRYAB content, which stabilizes SMAD4 in this compartment, SMAD4 localization should also be affected by RE. We analyzed this via F‐IHC on human muscle biopsies taken pre‐ and post‐RE (Fig. 6A). RE reduces nuclear SMAD4 content in both fiber types (Fig. 6C). Generally, SMAD4 content was higher in type I than in type II fibers, both pre‐ and post‐RE (Fig. S2C). Additionally, sarcoplasmic SMAD4 content was also reduced after RE (Fig. 6D), corresponding to the model proposed by Bellaye *et al*. [23], where monoubiquitinated nuclear SMAD4 is degraded in the cytosol upon nuclear export.

TGF‐β1 and myostatin have been reported to regulate cell size as well by reducing the activity of the growth‐promoting mTOR cascade [1, 17, 18]. Ultimately, to determine whether RE intervenes in TGF‐β signaling by modulating CRYAB and SMAD4 nuclear localization, the activation of key members of anabolic signaling was analyzed pre‐ and post‐RE via WB (Fig. 6A). We determined a significant upregulation of mTOR phosphorylation at serine 2448, as well as of the rpS6 at serine 235/236 in response to RE (Fig. 6E).

## Discussion

In SkMFs, both CRYAB and SMAD4 play well‐established roles in homeostatic maintenance of skeletal muscle. CRYAB contributes to the proteostasis of cytosolic and cytoskeletal proteins under homeostatic stress [28], such as mechanically induced strain [37, 38, 39, 40], while SMAD4 mediates TGF‐β signaling [11] affecting muscle anabolism and repair [1, 17]. However, no cooperative function of these proteins in SkMFs has been previously reported, nor has the nuclear localization of CRYAB been described in human SkMFs. Here, we show that CRYAB is present in nuclei of human SkMFs, where it colocalizes with SMAD4. We further demonstrate that RE results in the reduction of nuclear CRYAB, which is supposed to be triggered by its phosphorylation at Ser59. Together with CRYAB, RE decreases SMAD4 in the nuclei and additionally in the cytosol, with a concurrently increasing phosphorylation of key targets of protein synthesis.

Previous reports of nuclear CRYAB localization have been limited to animal studies in glial cells [61] and *in vitro* experiments using cell lines such as HeLa [45], C2C12 [41, 46], and others like retinoblastoma and fibroblasts [43]. In C2C12 myoblasts, nuclear CRYAB [41, 46] has been observed specifically in proliferating myoblasts, but not in differentiated myotubes, which is consistent with our findings. Moreover, we show that nuclear CRYAB content decreases at an early stage of the differentiation process.

Interestingly, on the one hand, CRYAB overexpression in C2C12 cells (only cytoplasmic CRYAB was assessed, not nuclear) was reported to enhance proliferation and to impair cell cycle exit upon differentiation induction [46]. On the other hand, however, wild‐type C2C12 cells exhibit a gradual, up to 10‐fold increase in cytoplasmic CRYAB levels during differentiation [46, 62, 63], suggesting an essential role in myogenesis or later stages of differentiation. At first glance, these two results seem contradictory. However, assuming CRYAB's function in the differentiation process, our results may clarify the controversy between an inhibitory function on differentiation upon overexpression of CRYAB on the one hand and an increase in CRYAB levels observed upon differentiation of wild‐type C2C12 cells on the other. Our findings indicate that differentiation is regulated not just by CRYAB abundance, but also by its precise spatial distribution within the cell. Specifically, the presence of nuclear CRYAB in myoblasts and its early decrease upon differentiation initiation shown by us, coupled with a cytosolic increase [46, 62, 63], suggests a shift in its function from a yet unknown nuclear role in proliferating myoblasts to a cytoplasmic role in differentiation and myotube maturation.

Intriguingly, although CRYAB is absent from the nuclei of mature myotubes, we detected its presence in the nuclei of human and rat SkMFs, suggesting that nuclear localization of CRYAB is a conserved feature of adult mammalian skeletal muscle. Notably, its subnuclear localization was spatially associated with serine/arginine‐rich splicing factor SC35 (SC‐35) nuclear speckles (Fig. S2D), consistent with prior studies conducted in cell cultures, including C2C12 and HeLa cells [41, 43, 64]. However, the exact function of CRYAB within these nuclear speckles remains not fully understood. Recent works in fibroblasts suggest a novel nuclear role for CRYAB associated with TGF‐β signaling. Specifically, SMAD4 nuclear abundance is regulated by the E3 ubiquitin ligase TIF1γ, which promotes its monoubiquitination, leading to SMAD4 dissociation from the SMAD2/3 complex, nuclear export, and subsequent proteasomal degradation in the cytosol [19, 20, 21]. Bellaye *et al*. [23] demonstrated that CRYAB overexpression in fibroblasts sequestrates SMAD4 in the nucleus, whereas CRYAB silencing enhances its nuclear export. They proposed a model in which CRYAB interferes with TIF1γ‐mediated SMAD4 monoubiquitination upon TGF‐β1 stimulation, thereby promoting SMAD4 nuclear retention and profibrotic activity.

In resting human SkMFs, we observed colocalization of CRYAB and SMAD4, suggesting a potential interaction within this tissue as well. However, in SkMFs, TGF‐β signaling serves a different role than in fibroblasts, where it primarily drives fibrosis [65]. In muscle fibers, TGF‐β1 [8, 9], and especially myostatin [6, 7], act as negative regulators of fiber size. While in disease states like cancer, heart failure or HIV, both candidates contribute to the pathogenesis of muscle wasting; under physiological conditions, they act more as a “homeostatic brake,” maintaining the balance between muscle protein synthesis and degradation, preventing excessive muscle growth [66]. Conversely, RE is a well‐established stimulus for muscle growth, activating the AKT/mTOR‐pathway and promoting protein synthesis [2, 3, 4].

**Fig. 7:**
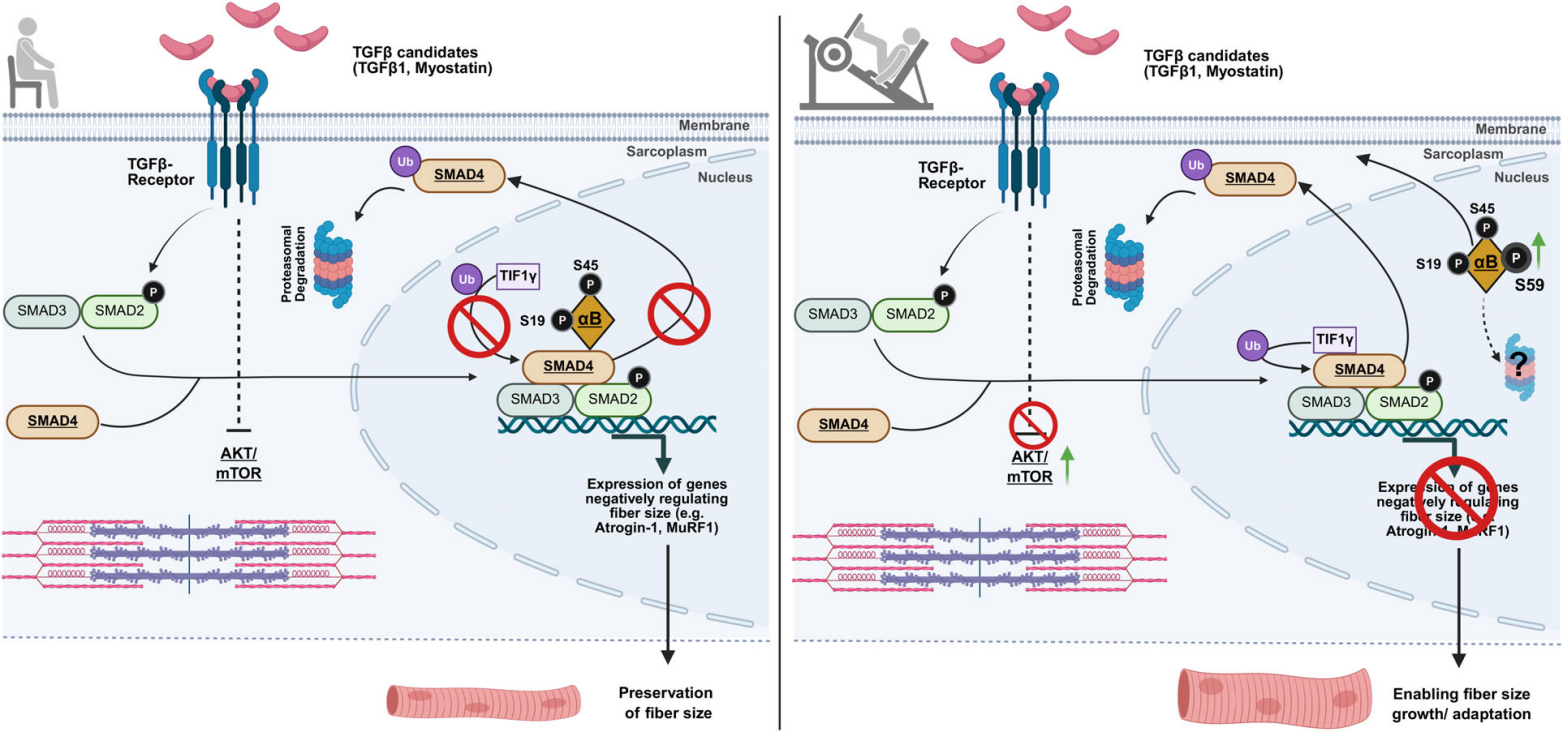
Hypothesized mechanism of CRYAB/SMAD4 interaction in the nuclei of skeletal muscle fibers (SkMFs) at rest and following resistance exercise (RE). In healthy individuals, who are physically active on a daily basis (left panel), TGF‐β1 and myostatin are continuously expressed, helping to maintain a balance between SkMF growth and atrophy, thereby maintaining muscle mass homeostasis. Canonically, the ligands bind to their TGF‐β receptor, which mediates signaling through the phosphorylation and activation of SMAD2/SMAD3. Next, the latter form a complex with SMAD4, which then translocates into the nucleus, where it binds DNA and initiates the transcription of negative regulators of fiber size like Atrogin‐1 and MuRF1. Furthermore, TGF‐β1 and myostatin inhibit the AKT/mTOR‐axis, which is a key player in protein synthesis by an as yet unknown mechanism. Nuclear CRYAB, which is constitutively phosphorylated at two of its three main sites, namely serine 19 and serine 45 (S19, S45), facilitates this transcription. Specifically, CRYAB protects SMAD4 from TIF1γ‐induced monoubiquitination, which would otherwise disrupt the transcription complex by leading to translocation of SMAD4 into the cytosol and subsequent proteasomal degradation. This disruption is hypothesized to be triggered by RE (right panel). Our findings show that RE leads to a reduction in nuclear CRYAB, either by its proteasomal degradation in the nucleus or, more likely, due to its export, which might be triggered by the phosphorylation at serine 59 (S59). Alongside CRYAB, SMAD4 levels decrease in both the nucleus and cytosol, accompanied by an induction of the AKT/mTOR‐axis via its relieved prior inhibition by TGF‐β signaling. From these findings, we derive that the loss of CRYAB facilitates SMAD4 monoubiquitination by TIF1γ, leading to its nuclear export and subsequent proteasomal degradation in the cytoplasm. This interrupts the expression of negative regulators of muscle mass and releases mTOR‐pathway activation, thereby supporting muscle adaptation and hypertrophy. Created in BioRender. Jacko, D. (2026) https://BioRender.com/4mn7fuz

As proof‐of‐concept in our acute RE setting, we confirmed the increased phosphorylation of key components of the mTOR‐pathway post‐RE (Fig. 6E). Importantly, we simultaneously observed a reduction in nuclear CRYAB and SMAD4 levels, alongside a decrease in cytosolic SMAD4. These findings suggest that SMAD4 is either degraded in the cytosol following nuclear export or that its expression is downregulated post‐exercise. This aligns with the model proposed by Bellaye *et al*. [23] for nuclear CRYAB/SMAD4 interaction in fibroblasts when adapted to SkMFs (Fig. 7). Based on this, we hypothesize that the RE‐induced decline in nuclear CRYAB may allow TIF1γ to ubiquitinate SMAD4, thereby destabilizing its interaction with SMAD2/3, promoting nuclear export, and facilitating cytosolic proteasomal degradation. This dissociation of the SMAD transcriptional complex could, in turn, downregulate the expression of negative regulators of muscle mass, thereby releasing the “homeostatic brake” and shifting the balance toward muscle protein synthesis and hypertrophy, which is initiated in the post‐exercise phase [2, 4, 67].

CRYAB phosphorylation is crucial for its function [33, 34, 35, 36]. While its role in the cytosol has been well‐characterized in both cell culture and human exercise studies, its significance within the nucleus remains unclear. In resting human SkMFs, we found CRYAB phosphorylated at Ser19, Ser45, and to a low degree at Ser59. Following RE, Ser59 phosphorylation increased, Ser45 phosphorylation decreased, and total CRYAB levels declined. The functional relevance of CRYAB phosphorylation in the nucleus remains a subject of debate. While it is established that nuclear CRYAB exists in a phosphorylated state [43, 44, 45, 61], its role, especially in its nuclear import/export, is unresolved. Van den Ijssel *et al*. [64] reported that phosphorylation does not regulate CRYAB nuclear localization in glioblastoma cells (U‐373 MG). Conversely, others demonstrated that pSer19 [44] and/or pSer45 [44, 45, 61] are required for nuclear speckle organization in HeLa and glial cells, while pSer59 was suggested to be critical for its nuclear import in HeLa cells [45]. However, conflicting findings indicate that pSer59 is exclusively cytosolic in oligodendrocytes [61], suggesting cell type specificity in CRYAB regulation. This is supported by the fact that pSer45 was detected in the nuclei of oligodendrocytes, but not in either the nuclei or cytosol of astrocytes [61].

Our findings in human SkMFs rather suggest that pSer59 is associated with nuclear export or degradation of CRYAB, as nuclear total CRYAB levels declined post‐RE while pSer59 levels increased at the same time. CRYAB is phosphorylated at serine 59 by MK2 (MAP mitogen‐activated protein kinase‐activated protein kinase 2), which in turn is activated by phosphorylation via p38 MAPK (Mitogen‐activated protein kinase 11–14) [34, 68, 69, 70]. Notably, both kinases are known to shuttle between the cytosol and nucleus in a dependent manner. Cytosolic p38 translocates to the nucleus upon its phosphorylation [71] while MK2 shuttles between the nucleus and cytoplasm via an intrinsic nuclear localization signal and a p38‐induced phosphorylation‐regulated nuclear export signal [72]. Thus, spatially it is plausible that CRYAB undergoes Ser59 phosphorylation within the nucleus, facilitating its nuclear depletion. Furthermore, p38 as well as MK2 phosphorylation were observed in SkMFs following muscle contraction and RE, at least in the cytosol [73, 74, 75, 76, 77]. However, to date there is no data on the regulation of both kinases specifically in the nuclei of skeletal muscle fibers. This would be important regarding the role of _p_CRYAB^Ser59^ in nuclear localization and should therefore be investigated in follow‐up studies.

In contrast to pSer59, nuclear pSer45 declined post‐RE along with total CRYAB levels. At first glance, this supports previous findings, suggesting that pSer45 is required for nuclear speckle organization or CRYAB retention [44, 45, 61]; thus, the observed dephosphorylation could be necessary for nuclear export or decrease of the protein. However, when normalizing pSer45 to total CRYAB levels, no significant difference between pre‐ and post‐RE remained, indicating that the reduction in pSer45 may be due to protein loss rather than dephosphorylation.

The mechanism underlying RE‐induced nuclear CRYAB depletion remains uncertain. Two possibilities include: (a) nuclear export and (b) nuclear degradation. If CRYAB were exported from the nucleus, a corresponding increase in cytosolic levels might be expected. However, we did not observe a significant cytosolic accumulation in this or previous studies [37, 38], which could argue against export as the primary mechanism. Nonetheless, prior studies indicate that CRYAB shuttles between the cytosol and nucleus. Adhikari *et al*. [41] reported that CRYAB translocates to the nucleus upon heat stress in C2C12 myoblasts and relocalizes to the cytosol upon recovery. Additionally, CRYAB nuclear export is impeded by leptomycin B‐mediated inhibition of exportin‐1, suggesting that passive diffusion in its monomeric state is unlikely. Notably, CRYAB lacks classical nuclear localization or export sequences [42], implying a non‐canonical transport mechanism. For import, the SMN complex has been shown to be involved [42]. Similar non‐classical nuclear export is conceivable, but its precise mechanism remains unknown.

Degradation of CRYAB within the nucleus is another potential mechanism for its decline post‐RE. There is not much literature on CRYAB degradation, but the ubiquitin proteasomal system seems to be the main route and phosphorylation of all three sites of the protein is necessary for this process [78]. However, further research is needed to determine whether the degradation of CRYAB in the nucleus contributes to RE‐induced CRYAB loss in this compartment.

In summary, we demonstrate that CRYAB is present in the nuclei of human SkMFs, where it colocalizes with SMAD4. Acute RE leads to the phosphorylation of CRYAB at serine 59, which induces its reduction in or export from nuclei. Along with CRYAB, SMAD4 is also reduced post‐RE in the nuclei and additionally in the cytoplasm. Simultaneously, anabolic signaling pathways are activated. Based on our findings, we adapted the CRYAB/SMAD4 interaction model for fibroblasts originally proposed by Bellaye *et al*. [23], to SkMFs (Fig. 7).

We conclude that the RE‐induced reduction in nuclear CRYAB and SMAD4, along with cytosolic SMAD4 depletion, likely represents a temporary mechanism to attenuate TGF‐β/myostatin signaling, thereby supporting skeletal muscle anabolism and adaptation after RE.

## Materials and methods

### Cell experiments

#### Cell culture

Murine C2C12 cells, obtained through serial passage of myoblasts cultured from the thigh muscle of C3H mice, were provided by Helen M. Blau (Stanford University). Cells were cultured at 37 °C and 5% CO_2_ in Dulbecco's modified Eagle medium (Invitrogen, Carlsbad, CA, USA) enriched with 20% fetal calf serum (FCS; Gibco, Invitrogen, USA), 1% penicillin/streptomycin solution (Invitrogen, USA), 4 mm glutamine (Invitrogen, USA), 1.5 g/L sodium bicarbonate (Invitrogen, USA), and 1 mm sodium pyruvate (Invitrogen, USA). The medium was changed every 48 h or with every new passage. All experiments were performed with mycoplasma‐free C2C12 cells.

#### Transfection

C2C12 myoblasts were seeded on a cover glass (BR723015‐100EA, Merck, Darmstadt, Germany) in gelatin‐coated six‐well plates either with or without coverslips, depending on the intended downstream application: Reverse transcription quantitative polymerase chain reaction (RT‐qPCR), western blot (WB), and fluorescence immunohistochemistry (F‐IHC). Cells were plated at a density of 1.63 × 10^4^ cells/mL for RT‐qPCR and WB analysis, and 1.56 × 10^2^ cells/mL for F‐IHC.

For siRNA‐mediated knockdown of CRYAB, cells were transfected using Lipofectamine RNAiMAX (Invitrogen, USA) according to the manufacturer's protocol. Briefly, 150 μL Opti‐MEM (Invitrogen) containing 4 μL Lipofectamine RNAiMAX was combined with 150 μL Opti‐MEM containing 2.4 μL siRNA (Ref # 1605828, CryabMSS203276, Invitrogen) and added to each well. After a 15‐min incubation at room temperature, 2 mL of the respective cell suspension was added per well. Cells were incubated for 48 h at 37 °C before further analysis.

#### Differentiation initiation

For differentiation initiation, 1 × 10^4^ C2C12 myoblasts were cultured on a cover glass (Merck, Germany) in gelatin‐coated six‐well plates. Upon reaching 90–100% confluence, the cells were washed three times with phosphate‐buffered saline (PBS), and the medium was replaced with differentiation medium containing 10% horse serum (Invitrogen, USA) instead of 20% FCS.

#### Animal experiments

Non‐ovariectomized female Wistar rats (Crl:WI; 5–6 weeks old; Charles River Laboratories, Wilmington, MA, USA) were included in the study. Prior to the intervention, all animals underwent a 7–10‐day acclimatization period under standardized environmental conditions: 22 ± 2 °C ambient temperature and a 12‐h light/dark cycle (lights on at 7:00 AM and off at 7:00 PM). Animals had *ad libitum* access to water and a phytoestrogen‐free chow (V1554; Sniff) throughout the study. Following acclimatization animals were housed in groups of three per cage (Sealsafe® NEXT, Tecniplast S.p.A., Buguggiate, Italy). At the end of the intervention, animals were euthanized, and tissue and blood samples were collected for subsequent biochemical and histological analyses. The collected muscles were cleaned of blood and non‐muscular tissues, embedded in TissueTek (Sakura Finetek, Zoeterwoude, Netherlands), frozen in liquid nitrogen‐cooled isopentane, and stored at −80 °C before further analysis.

All procedures were conducted in accordance with national guidelines for the care and use of laboratory animals and were approved by the Landesamt für Natur, Umwelt und Verbraucherschutz North Rhine‐Westphalia (protocol number: 81‐02.04.2022.A306; date of approval: 09 August 2022).

### Human experiments

#### Ethics approval and subjects

The protocols used in the present study were approved by the ethics committee of the German Sports University Cologne (application number: 005/2018) and comply with the declaration of Helsinki. All subjects were informed verbally and in written form about the purpose of the study and risks involved before signing the declaration of consent to participate in this study.

We recruited healthy, active, but not exceptionally resistance‐ or endurance‐trained subjects (*n* = 10: 7 male, 3 female; age: 25.1 ± 3.4 yrs; height: 177.3 ± 9.0 cm; weight: 74.3 ± 13.5 kg) via an online call for volunteers that was posted on the website of the German Sport University Cologne. All subjects performed a lower‐body RE. The RE was performed on a leg extension machine and a leg press machine (both Gym80, Gelsenkirchen, Germany). Sample collection took place from June 2020 to June 2022.

#### Resistance exercise protocol

Each session began with a standardized warm‐up consisting of 5 min cycle ergometry at a 1 W/kg body weight load, followed by a device‐specific warm‐up involving 10 repetitions with a load set at 70% of the 10‐repetition maximum (RM). The main RE was designed as follows: First, leg extensions (Gym80 Signum)—two sets of 4 repetitions performed with the 4 RM, two sets of 8 repetitions with the 8 RM, and one additional set of 8 repetitions with 70% intensity—in which the concentric phase was performed bilaterally and the eccentric phase alternatingly with one leg only—and two final sets of 16 repetitions with the 16 RM. After that, the same protocol was performed on a leg press (MANG Sportgeräte), excluding the last two sets of 16 reps.

For both exercises, the contraction pattern was 1 s concentric and 2 s eccentric with 0.5 s for the change of the direction of motion. Rest intervals were 2 min between sets and 3 min between exercises.

The RT concluded with 2 sets of 10 drop‐jumps from a height of 60 cm, followed by a stair descent over 182 stairs with a height of 18 cm each, where two steps were taken at once.

#### Muscle biopsies

Biopsy collection took place in the Outpatient Clinic for Sports Traumatology at the German Sports University Cologne (Cologne, Germany). In total, two biopsies were taken from the vastus lateralis of each subject, one from each leg, using the Bergström‐needle technique.

The subjects were instructed to fast overnight before biopsy days and to consume a standardized meal consisting of a nutrient drink (Fresubin Vanille, Fresenius; 20 g protein, 24.8 g carbs, 13.4 g fat, 1260 kJ) 3 h before biopsies. All subjects were allowed to drink water *ad libitum*.

The first biopsy was taken under resting conditions (pre) in the morning between 8 AM and 10 AM. RE and post‐exercise biopsies were performed approximately one week after pre‐exercise biopsies, with the post‐biopsies taken one hour after the completion of the RE, also in the morning between 7 AM and 10:30 AM.

The collected muscle samples were cleaned of blood and non‐muscular tissues, embedded in TissueTek (Sakura Finetek, Zoeterwoude, Netherlands), frozen in liquid nitrogen‐cooled isopentane, and stored at −80 °C before further analysis.

### Analysis

#### RNA isolation and RT‐qPCR

For RNA isolation, cells were lysed in 1 mL TRIzol reagent (Ambion, Carlsbad, CA, USA) per well, and phase separation was performed by adding 0.2 mL chloroform. After centrifugation (16 595 ***g***, 15 min, 4 °C), the aqueous phase was transferred, and RNA was precipitated with isopropanol (0.5 mL per 1 mL TRIzol). The RNA pellet was washed with 75% ethanol, air‐dried, and resuspended in 15 μL RNase‐free water. RNA concentrations were determined using a NanoDrop 1000 Spectrophotometer (Thermo Fisher Scientific Waltham, MA, USA) by measuring absorbance at 260 nm.

RT‐qPCR was performed using the PerfeCTa^®^ SYBR^®^ Green FastMix^®^ Low Rox Kit (84073; QuantaBio Beverly, MA, USA) in a 96‐well plate format (Agilent Mx3005P, Santa Clara, CA, USA). Primer sequences for target genes and housekeeping genes are listed in Table S1. Reactions contained 10 μL SYBR Green, 1 μL primer mix, 7 μL RNase‐free water, and 2 μL cDNA per well, resulting in a total volume of 20 μL. No‐template controls (NTCs) were included for each primer pair.

RT‐qPCR cycling conditions were as follows: initial denaturation at 95 °C for 2 min, followed by 40 cycles of 95 °C for 10 s, 60 °C for 15 s and 72 °C for 30 s. Melting curve analysis was performed to confirm amplification specificity.

### Protein isolation

#### Cell harvesting

Cells were harvested by trypsinization and centrifuged at 115 ***g*** for 10 min at 21 °C. After removing the supernatant, cell pellets were washed twice with PBS, lysed in 100 μL urea lysis buffer (4 m Urea, 20% SDS, 1 m DTT, 1.5 m Tris, Glycerol), and homogenized using a Precellys Lysing Kit and a Precellys Evolution Touch homogenizer (both Bertin Technologies, France) for 3 Cycles (20 s) at 4500 rpm, with 2 min rest intervals. Samples were incubated at room temperature for 30 min and centrifuged at 19 645 ***g*** for 15 min at 21 °C.

#### Subcellular protein fractionation of human muscle tissue

Frozen muscle samples were sectioned into 20 μm thick flakes using a cryostat (Leica CM 3050 S, Leica Microsystems, Nußloch, Germany) and transferred into pre‐chilled 2 mL tubes (Sarstedt, Nümbrecht, Germany) containing homogenization beads (Precellys Lysing Kit, #P000918‐LYSK0‐A, Bertin Technologies, Montigny le Bretonneux, France). Subcellular fractionation was performed using differential buffer extraction on ice, according to the manufacturer's protocol (Subcellular Protein Fractionation Kit for Tissues, 87790, Thermo Fisher, USA) with minor adaptations.

Briefly, Cytoplasmic Extraction Buffer (CEB), Membrane Extraction Buffer (MEB), and Nuclear Extraction Buffer (NEB), each supplemented with protease/phosphatase inhibitors (#78429; 1 : 100; Thermo Scientific, Waltham, MA, USA) and PMSF (#P7626‐1G; 1 : 100; Sigma Life Science, St. Louis, MO, USA), were sequentially added to the pellet with intermittent vortexing and incubation steps. Chromatin‐associated proteins were released using NEB supplemented with CaCl_2_ and Micrococcal Nuclease (NEB+), followed by extraction of insoluble proteins using 4 m Urea Lysis Buffer and mechanical homogenization (Precellys, 4500 rpm, 3 × 20 s). After each extraction step, samples were centrifuged (CEB and MEB: 3000 × ***g***; NEB: 500 × ***g***; NEB+ and Urea LB: 16 000 × ***g***) and supernatants were collected for downstream analyses.

Additionally, muscle tissue was homogenized and lysed using a Triton X‐100‐based buffer (#9803; Cell Signaling, Beverly, MA, USA) containing protease and phosphatase inhibitors (Thermo Scientific, USA) and PMSF (Sigma Life Science, USA). A Precellys lysing kit and Precellys Evolution (see above) were used for homogenization, applying 35 μL of buffer per 1 mg of tissue. The samples were homogenized for 3 cycles of 15 s at 5500 rpm with 2‐min resting intervals on ice. After 15 min of incubation on ice, the samples were centrifuged at 19 645 ***g*** for 10 min, and the supernatant was collected.

### Western blot analysis

Western blotting was carried out according to a previously documented method [79]. Protein concentration of cell and human muscle lysates were determined via a Lowry test kit (BioRad Laboratories GmbH, Munich, Germany). Before analysis, each sample was diluted to a protein concentration of 1.5 mg/mL and suspended in a 4× Laemmli buffer (#1610747; BioRad, Germany). Each sample, except the pellet‐fractions, was heated at 95 °C for 5 min.

Equal amounts of protein (15 μg) for each sample were separated in an 18‐well, 4–12% BIS‐TRIS Gel using a gel‐casting system compatible with MOPS electrophoresis buffer. All used equipment and buffers were products of BioRad. After electrophoresis, the gel was transferred to a polyvinylidene difluoride (PVDF) membrane (GE Healthcare Life Science, Amersham, UK) by semidry blotting (Trans Blot Turbo, Bio‐Rad, Hercules, CA, USA) for 40 min (1.2 A, at a maximum of 25 V). After transfer completion, equal sample loading and transfer were checked by staining the PVDF membrane with Ponceau S.

Membranes were blocked for one hour at room temperature in 5% nonfat dry milk dissolved in tris‐buffered saline supplemented with 0.1% Tween20 (TBST) before being incubated with primary antibodies dissolved in 5% bovine serum albumin in TBST overnight at 4 °C. Then, membranes were washed in TBST and subsequently incubated with secondary antibodies diluted in TBST containing 5% nonfat dry milk for one hour at room temperature. After washing again, membranes were incubated for 3 min with an enhanced chemiluminescence assay (ECL‐Kit, GE Healthcare Life Science, Amersham, UK) and automatically captured (ChemiDoc MP, Bio‐Rad, Hercules, CA, USA). All used primary and secondary antibodies are listed in Table S2.

### F‐IHC staining

#### Cell experiments

Cells were fixed directly onto slides by removing the growth medium and washing twice with 2 mL PBS per well. Subsequently, 1.5 mL of ice‐cold 1 : 1 Methanol‐Acetone solution was added for 2 min at room temperature. After removal of the fixing solution, cells were washed three times for 5 min each with 2 mL PBS per well.

Unless stated otherwise, all washing steps consisted of three short rinses (10–15 s) followed by two 15 min washes with tris‐buffered saline (TBS; 150 mm NaCl, 10 mm Tris–HCl, pH 7.6).

Cells were blocked with 5% bovine serum albumin (BSA) in TBS for 60 min at room temperature. Primary antibodies were diluted in 0.8% BSA in TBS and incubated overnight at 4 °C. After washing, appropriate secondary antibodies diluted in TBS were applied for 60 min at room temperature in the dark. All used primary and secondary antibodies are listed in Table S2. If required, double staining was performed by applying an additional primary antibody and processing accordingly as described before. For membrane staining, Wheat Germ Agglutinin (1 : 500, Thermo Fisher, USA) was applied for 15 min. Finally, a coverslip was mounted on the Polysine microscope slides (VWR) with Aqua‐Poly/Mount (Polysciences, Germany).

#### Human experiments

Frozen muscle samples were consecutively cut into 7 μm thick cross‐sections using a cryo‐microtome (Leica CM 3050 S, Leica Microsystems, Germany) and mounted on Polysine microscope slides. Sections were air‐dried, fixed in pre‐cooled acetone (−20 °C) for 8 min, blocked with 5% BSA in TBS for 1 h, and stained following the same procedure as described for cell samples.

Subsequently, nuclei were counterstained with DRAQ5 (1 : 1500) for 6 min. If applicable, membranes were stained using Wheat Germ Agglutinin (1:  800 in TBS) for 30 min. Finally, slides were mounted with Aqua‐Poly/Mount (Polysciences Europe GmbH, Hirschberg an der Bergstraße, Germany) and covered with a coverslip (VWR International).

To confirm antibody specificity, control sections were incubated with 0.8% BSA without primary antibodies and processed identically. All time points of a given subject were mounted on the same slide, and all samples were stained in a single batch using identical antibody dilutions and incubation times to minimize variability in staining efficiency.

#### Microscopy and image analysis

Images from all experiments were acquired using a laser scanning confocal microscope (LSM 510 Meta, Zeiss, Jena, Germany) equipped with a Plan‐Apochromat 40×/1.0 objective lens. Microscope settings, including software parameters and laser configurations (helium‐neon 555 nm, argon multi‐line 488 nm, and 633 nm; Lasos Lasertechnik, Jena, Germany), remained unchanged across all samples to ensure comparability.

#### Cell experiments

For each slide, eight randomly selected fields of view were imaged, aiming to obtain approximately 90 individual cells per condition (control and treated). Only isolated, non‐overlapping cells were selected to avoid signal interference from neighboring cells.

CRYAB expression was semi‐quantitatively assessed using optical densitometry in Fiji ImageJ2 software [80]. Entire cells were defined as regions of interest (ROIs) using the wand tool based on the Wheat Germ Agglutinin (WGA) signal (blue, 633 nm). Cell area was measured in the blue channel (633 nm), and mean fluorescence intensity (gray value) was determined in the red channel (555 nm) corresponding to CRYAB staining. Nuclear CRYAB expression was assessed by selecting nuclei as ROIs in the corresponding channel (Lamin A/C: green, 488 nm, DRAQ5: blue, 633 nm), with the intensity measured in the red channel (555 nm). Only fully visible cells and nuclei were included in the analysis.

For Differentiation initiation experiments approximately 250 nuclei per time point were analyzed.

#### Human muscle samples

For human muscle cross‐sections, five overview images of randomized areas were acquired using a 0.7× digital zoom for fiber type classification, followed by three higher magnification images (2× digital zoom) per overview for densitometric analysis of nuclear staining. Nuclei were selected as ROIs in the blue channel (DRAQ5) using the wand tool, and fluorescence intensity of the target protein was measured. Additionally, each nucleus was classified as intracellular or extracellular based on its localization relative to the membrane. Approximately 115 intracellular nuclei per time point were analyzed.

For densitometric analysis of cytosolic staining, each fiber was selected as ROI in the membrane channel using the wand tool, and fluorescence intensity of the target protein was measured [80]. Approximately 120 fibers per time point were analyzed.

Fiber type of the measured cytosolic regions and nuclei was determined by matching corresponding fibers in the myosin heavy chain type I‐stained cross‐sections.

### Statistics

For all quantitative data from F‐IHC and WB experiments, mean, standard deviation (SD), and standard error of the mean (SEM) were calculated for treated and control groups in cell experiments and for each subject and time point in human samples. Normal distribution was assessed using the Kolmogorov–Smirnov test. As data sets were normally distributed, unpaired or paired *t*‐tests were performed to determine statistical significance (specification is noted in figure captions). For differentiation initiation experiments, statistical analysis was performed using the Kruskal–Wallis test and Dunn's test for multiple comparisons with “Pre” as the control column. Gene expression from RT‐qPCR was analyzed using the 2^−ΔΔCt^ method with glyceraldehyde‐3‐phosphate dehydrogenase (GAPDH) as the internal reference gene.

All statistical analyses and figures were generated using graphpad Prism software (version 10.2.1; GraphPad Software, San Diego, CA, USA) with default settings. The exact number of biological replicates (*n*) for each assay is reported in the figure legends.

## Conflict of interest

The authors declare no conflict of interest.

## Author contributions

DJ conceived and supervised the study; SG conceived the human exercise intervention, DJ, KS and CD designed experiments; KS, CD and TK performed experiments; KS, CD, KB, and TK performed data analysis; DJ wrote the manuscript; PD, WB, SG and DJ provided reagents, WB, SG, PD, and KB made manuscript revisions, SG, WB obtained funding, KS, CD, TK, PD, KB, WB, SG, and DJ contributed by interpretation of data, approved the final version to be published and agreed to be accountable for all aspects of the work.

## Supporting information


**Table S1.** Primers sequences utilized for RT‐qPCR amplification.
**Table S2.** Antibodies used for Fluorescence‐Immunohistochemistry (F‐IHC) and Western Blot (WB).
**Fig. S1.** (A) Experimental procedure. CRYAB was silenced in C2C12 myoblasts via siRNA. 15 Transfected and control samples were subjected to RT‐qPCR (above). 16 Resting skeletal muscle samples were collected from human vastus lateralis and gastrocnemius of the 17 rat and subjected to subcellular fractionation into cytosolic, membrane, nucleoplasmic, chromatin18 bound, and pellet or cytoskeletal proteins, followed by western blot analysis (below). 19 (B) RT‐qPCR amplification plots for control and transfected samples using CRYAB and GAPDH primers 20 (*n* = 5). RT‐qPCR amplification plot of all loaded samples including those with GAPDH and CRYAB primer 21 mixes. GAPDH control and transfected (black bar); CRYAB control (magenta bar); CRYAB transfected 22 (green bar). (C) Representative Ponceau S staining images of subcellular fractions of human skeletal 23 muscle (*n* = 2) described in (A) (left) and gastrocnemius muscle of the rat (*n* = 3; right).
**Fig. S2.** (A) Experimental procedure. Human skeletal muscle biopsies were collected before (pre) 26 and one hour after (post) a single bout of resistance exercise (RE). Biopsies were cryo‐sectioned and 27 used for fluorescence immunohistochemistry (F‐IHC), then imaged with a laser scanning microscope 28 (LSM). (B) Fiber‐type specific quantification of nuclear total and phospho CRYAB signal (Ser19, Ser45 29 and Ser59) pre‐ and post‐RE (*n* = 10). Quantification of staining intensity of CRYAB in nuclei of type I 30 and type II muscle fibers. (C) Fiber‐type specific quantification of nuclear and cytosolic SMAD4 signal 31 pre‐ and post‐RE (*n* = 10). Quantification of staining intensity of SMAD4 in nuclei and cytosol of type I 32 and type II muscle fibers. Data are presented as mean fluorescence intensity and individual data points 33 are shown. Statistical analysis was performed using two‐tailed paired *t*‐tests. *P < 0.05; **P < 0.01; ***P < 0.001; ns = not significant. (D) Representative F‐IHC image of total CRYAB/SC‐35‐colocalization in 35 nuclei of human SkMFs at rest (*n* = 3). Staining: CRYAB (green), SC‐35 (magenta), nuclei (DRAQ5, blue). 36 Scale bars: 30 μm for overview image, 5 μm for zoomed‐in images.

## Data Availability

The data presented in this study are available on request from the corresponding author.
