# Supplementary material for: Resistance‐exercise‐induced stress intervenes in TGF‐β signaling by cooperatively downregulating nuclear αB‐crystallin and SMAD4 in human skeletal muscle fibers
Source: FEBS J. 2026 Feb 23;293(15):4624–44. doi: 10.1111/febs.70468 (PMC13440606; doi:10.1111/febs.70468)
Supplement: Supplementary file 1 [file FEBS-293-4624-s001.pdf]

Supplemental Data

Methods

Table 1. Primers sequences utilized for RT-qPCR amplification

Gene	Primer	Sequence 5'-3'
GAPDH	Forward	TGCGACTTCAACAGCAACTC
	Reverse	GCCTCTCTCTTGCTCAGTGTCC
CRYAB	Forward	ACACCGGACTCTCAGAGATG
	Reverse	GGACCTCAATCACGTCCCC
Myogenin	Forward	CCCATGGTGCCCAGTGAA
	Reverse	GCAGATTGTGGGCGTCTGTA
Myf5	Forward	CAGCCCCACCTCCAACTG
	Reverse	GCAGCACATGCATTTGATACATC

Table 2. Antibodies used for Fluorescence-Immunohistochemistry (F-IHC) and Western Blot (WB)

Primary Antibodies	Dilution	Host	Catalog #	Manufacturer
A4.951	1:50 (F-IHC)	Mouse	AB_528385	DSHB
$_{Total}$CRYAB (polyclonal)	1:100 (F-IHC)<br>1:1000 (WB)	Rabbit	NB120-13497	Novus
$_{Total}$CRYAB (monoclonal)	1:100 (F-IHC)	Mouse	ADI-SPA-222-F	Enzo Lifesciences
$_{p}$CRYAB^{Ser19}	1:100 (F-IHC)	Rabbit	ADI-SPA-225-E	Enzo Lifesciences
$_{p}$CRYAB^{Ser45}	1:100 (F-IHC)	Rabbit	ADI-SPA-226-F	Enzo Lifesciences
$_{p}$CRYAB^{Ser59}	1:100 (F-IHC)	Rabbit	ADI-SPA-227-F	Enzo Lifesciences
Dystrophin	1:75 (F-IHC)	Mouse	NCL- DYS2	Leica Biosystems
Lamin A/C	1:50 (F-IHC)	Mouse	MANLAC5[7F12]	DSHB
$_{Total}$mTOR	1:1000 (WB)	Mouse	4517S	Cell Signaling
$_{p}$mTOR^{Ser2448}	1:1000 (WB)	Rabbit	5536S	Cell Signaling
$_{Total}$rpS6	1:1000 (WB)	Mouse	2317S	Cell Signaling
$_{p}$rpS6$^{Ser235/236}$	1:1000 (WB)	Rabbit	4858S	Cell Signaling
SC-35	1:100 (F-IHC)	Mouse	S4045	Sigma
SMAD4	1:100 (F-IHC)	Mouse	MA5-15682	Thermo Fischer Scientific

Secondary Antibodies	Dilution	Host	Catalog #	Manufacturer
Alexa Fluor 488 anti mouse IgG	1:500 (F-IHC)	Goat	A11029	Thermo Fischer Scientific
Alexa Fluor 488 anti rabbit IgG	1:500 (F-IHC)	Goat	A11037	Thermo Fischer Scientific
Alexa Fluor 555 anti mouse IgG	1:500 (F-IHC)	Goat	A21424	Thermo Fischer Scientific
Alexa Fluor 555 anti rabbit IgG	1:500 (F-IHC)	Goat	A21429	Thermo Fischer Scientific
Alexa Fluor 633 anti mouse IgG	1:500 (F-IHC)	Goat	A21052	Thermo Fischer Scientific
Anti-mouse igG, HRP-linked	1:7500 (WB)	Goat	7076S	Cell Signaling
Anti-rabbit igG, HRP-linked	1:7500 (WB)	Goat	7074S	Cell Signaling

Results

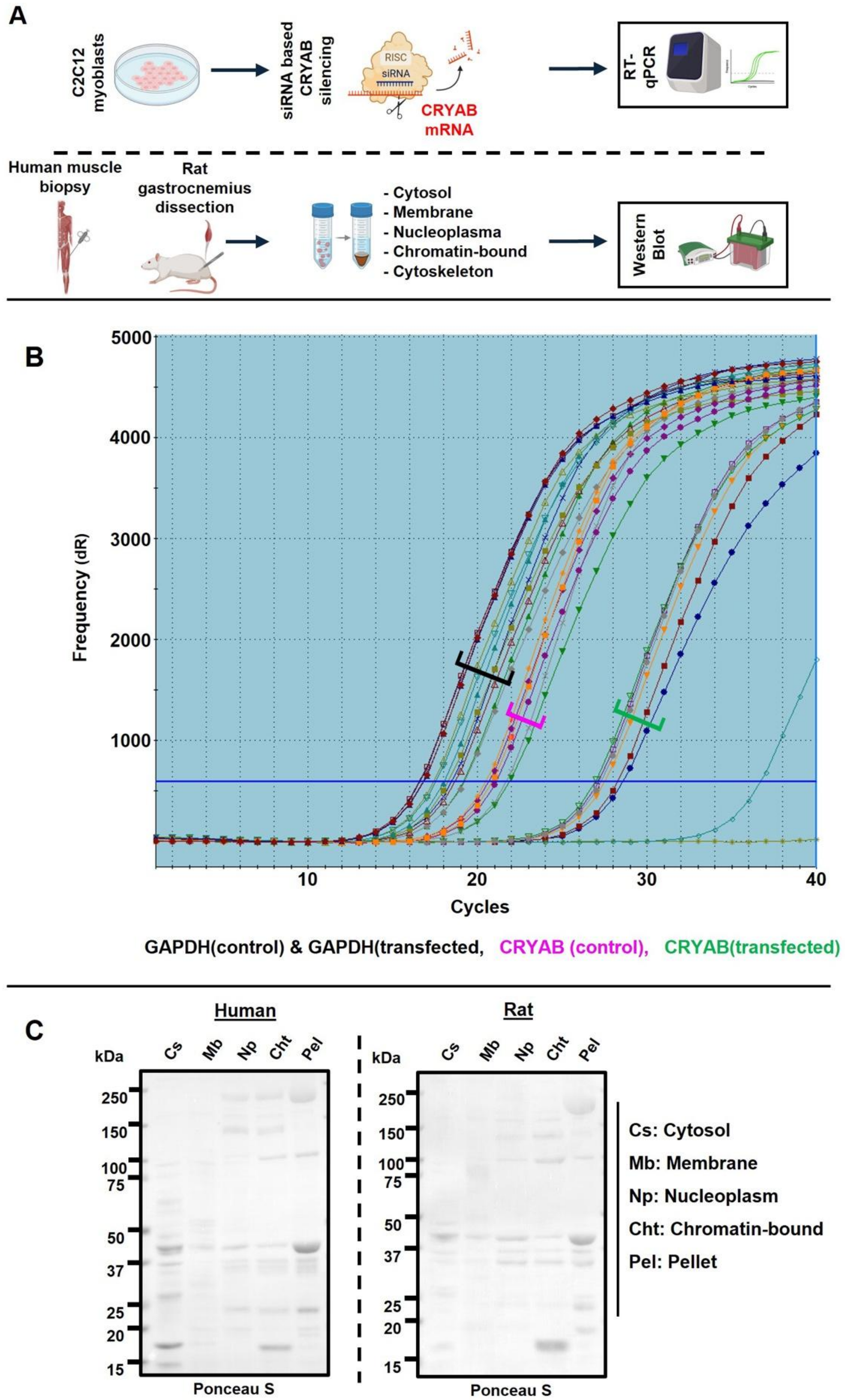

A
C2C12 myoblasts
siRNA based CRYAB silencing
RISC
siRNA
CRYAB mRNA
RT-qPCR
Human muscle biopsy
Rat gastrocnemius dissection
- Cytosol
- Membrane
- Nucleoplasma
- Chromatin-bound
- Cytoskeleton
Western Blot
B
Frequency (dR)
5000
4000
3000
2000
1000
Cycles
10
20
30
40
GAPDH(control) & GAPDH(transfected, CRYAB (control), CRYAB(transfected)
C
Human
Rat
kDa
Cs
Mb
Np
Cht
Pel
250
150
100
75
50
37
25
20
15
Ponceau S
Cs: Cytosol
Mb: Membrane
Np: Nucleoplasm
Cht: Chromatin-bound
Pel: Pellet

Figure S1: (A) Experimental procedure. CRYAB was silenced in C2C12 myoblasts via siRNA. Transfected and control samples were subjected to RT-qPCR (above).

Resting skeletal muscle samples were collected from human vastus lateralis and gastrocnemius of the rat and subjected to subcellular fractionation into cytosolic, membrane, nucleoplasmic, chromatin-bound, and pellet or cytoskeletal proteins, followed by western blot analysis (below).

(B) RT-qPCR amplification plots for control and transfected samples using CRYAB and GAPDH primers (n=5). RT-qPCR amplification plot of all loaded samples including those with GAPDH and CRYAB primer mixes. GAPDH control and transfected (black bar); CRYAB control (magenta bar); CRYAB transfected (green bar). **(C)** Representative Ponceau S staining images of subcellular fractions of human skeletal muscle (n=2) described in (A) (left) and gastrocnemius muscle of the rat (n=3; right).

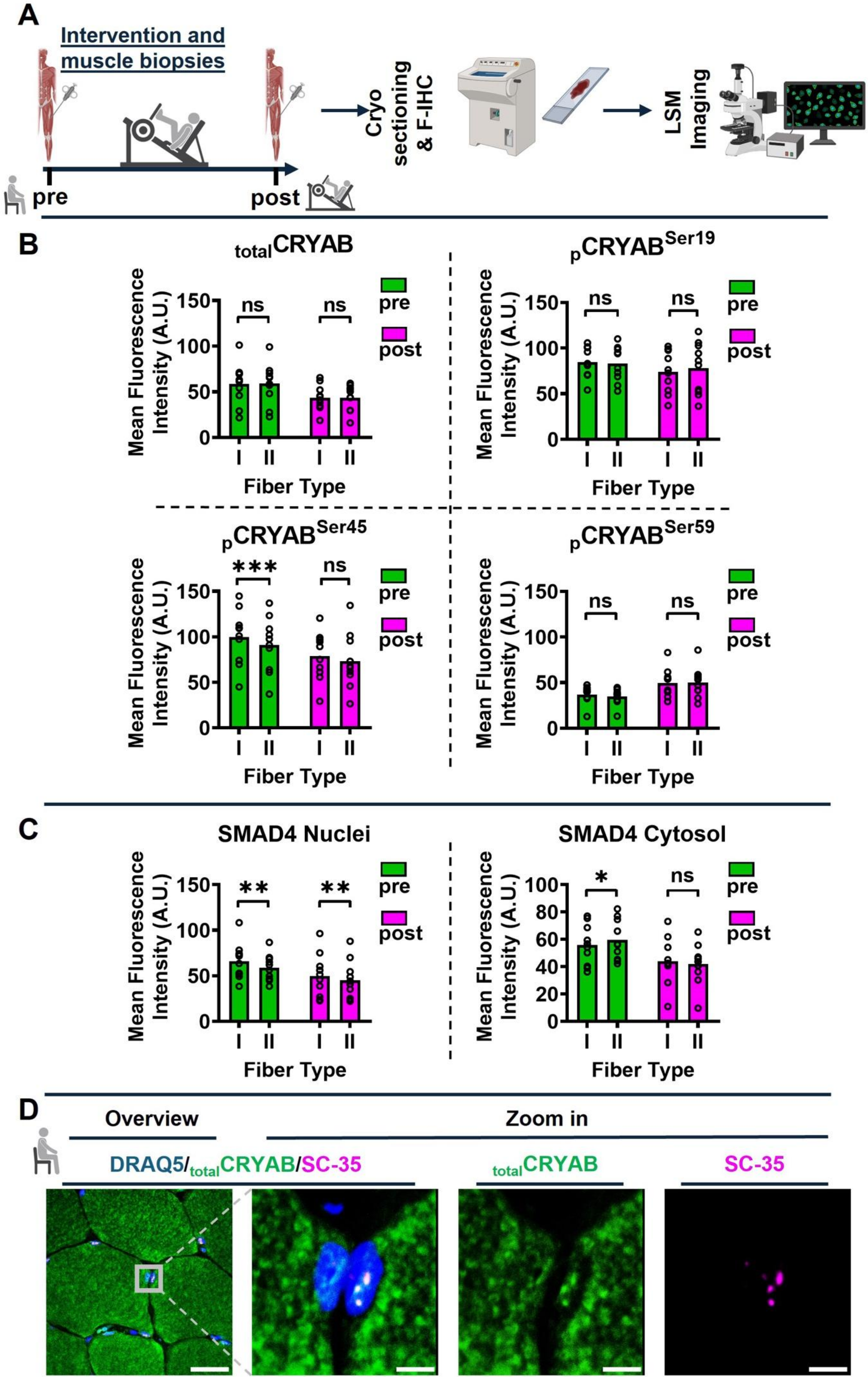
A
Intervention and muscle biopsies
pre
post
Cryo sectioning & F-IHC
LSM Imaging
B
totalCRYAB
pCRYAB Ser19
pCRYAB Ser45
pCRYAB Ser59
Mean Fluorescence Intensity (A.U.)
Fiber Type
pre
post
ns
***
C
SMAD4 Nuclei
SMAD4 Cytosol
**
*
D
Overview
Zoom in
DRAQ5/totalCRYAB/SC-35
totalCRYAB
SC-35

Figure S2: (A) Experimental procedure. Human skeletal muscle biopsies were collected before (pre) and one hour after (post) a single bout of resistance exercise (RE). Biopsies were cryo-sectioned and used for fluorescence immunohistochemistry (F-IHC), then imaged with a laser scanning microscope (LSM). **(B)** Fiber-type specific quantification of nuclear total and phospho CRYAB signal (Ser19, Ser45 and Ser59) pre- and post-RE (n=10). Quantification of staining intensity of CRYAB in nuclei of type I and type II muscle fibers. **(C)** Fiber-type specific quantification of nuclear and cytosolic SMAD4 signal pre- and post-RE (n=10). Quantification of staining intensity of SMAD4 in nuclei and cytosol of type I and type II muscle fibers. Data are presented as mean fluorescence intensity and individual data points are shown. Statistical analysis was performed using two-tailed paired t-tests. *: $p<0.05$; **: $p<0.01$; ***: $p<0.001$; ns = not significant. **(D)** Representative F-IHC image of total CRYAB/SC-35-colocalization in nuclei of human SkMFs at rest (n=3). Staining: CRYAB (green), SC-35 (magenta), nuclei (DRAQ5, blue). Scale bars: 30 µm for overview image, 5 µm for zoomed-in images.
